# Ligands of the peripheral benzodiazepine receptor induce apoptosis and cell cycle arrest in oesophageal cancer cells: involvement of the p38MAPK signalling pathway

**DOI:** 10.1038/sj.bjc.6601125

**Published:** 2003-07-29

**Authors:** A P Sutter, K Maaser, B Barthel, H Scherübl

**Affiliations:** 1 Medical Clinic I, Gastroenterology, Infectious Diseases, Rheumatology, University Hospital Benjamin Franklin, Free University of Berlin, Hindenburgdamm 30, 12200 Berlin, Germany

**Keywords:** peripheral benzodiazepine receptor, p38MAPK, gene expression, apoptosis, cell cycle, cDNA array

## Abstract

Specific ligands of the peripheral benzodiazepine receptor (PBR) are known to induce apoptosis and cell cycle arrest in oesophageal cancer cells. However, the underlying mechanisms are still unknown. Here, we investigated the transcriptional alterations and activation of protein kinases in response to PBR-specific ligands. Using cDNA arrays, we examined the transcriptional effects of the PBR-specific ligand FGIN-1-27 in two oesophageal cancer cell lines, KYSE-140 (squamous cell carcinoma) and OE-33 (adenocarcinoma). In oesophageal cancer cells, FGIN-1-27 induced extensive changes in the expression of genes involved in the regulation of apoptosis and cell cycle. Both in oesophageal cancer cell lines (KYSE-140, OE-33) we observed a strong upregulation of the growth arrest and DNA-damage-inducible genes, *gadd45* and *gadd153*, in response to PBR ligands. *gadd* genes are known to be induced by p38MAPK activation. Using Western blotting we detected a time- and dose-dependent phosphorylation of p38MAPK, which was found to be functionally involved in *gadd* induction, apoptosis, and cell cycle arrest. In conclusion, our data indicate that PBR-specific ligands cause apoptosis and cell cycle arrest by activation of the p38MAPK pathway and induction of *gadd45* and *gadd153*.

Originally, the peripheral benzodiazepine receptor (PBR) was described as another class of binding sites for benzodiazepines distinct from the central benzodiazepine receptor (CBR) (Braestrup and Squires, 1977). It was identified as a mitochondrial 18 kDa protein (McEnery *et al*, 1992). In contrast to CBR, PBR exhibits high affinity for the isoquinoline carboxamide PK 11195 and the indoleacetamide FGIN-1-27 (Kozikowski *et al*, 1993). Conversely, clonazepam binds with high affinity to CBR, but has extremely low affinity for PBR (Wang *et al*, 1984b). In the outer mitochondrial membrane, PBR is associated with the voltage-dependent anion channel and the adenine nucleotide translocator, all of which contribute to the formation of the mitochondrial permeability transition pore (McEnery *et al*, 1992).

Although present in all tissues to some extent, PBR is highly expressed in steroid-producing tissues (Beurdeley-Thomas *et al*, 2000). Besides its well-established function in the regulation of steroidogenesis (Papadopoulos *et al*, 1997), the abundance of PBR in cancers of the colon (Katz *et al*, 1990b; Maaser *et al*, 2002a), brain (Cornu *et al*, 1992), breast (Hardwick *et al*, 1999), ovary (Katz *et al*, 1990a), and liver (Venturini *et al*, 1998) suggests an additional role in tumorigenesis. The proliferation of breast cancer (Beinlich *et al*, 1999; Carmel *et al*, 1999), melanoma (Landau *et al*, 1998), Leydig's cell tumour (Garnier *et al*, 1993), astrocytoma (Neary *et al*, 1995), colorectal (Maaser *et al*, 2001), and oesophageal carcinoma (Sutter *et al*, 2002) was shown to be inhibited by PBR-specific ligands. In haematopoetic and epithelial cells, the antiproliferative effects of PBR-specific ligands were mediated by the induction of apoptosis and cell cycle arrest (Tanimoto *et al*, 1999; Maaser *et al*, 2001; Sutter *et al*, 2002). However, the underlying mechanisms of PBR-ligand-mediated apoptosis are far from being understood. Several reports have been published on the potential mechanisms contributing to PBR-ligand-mediated apoptosis. Recently, we have shown that mitochondrial membrane permeabilisation is a necessary and early step in PBR-ligand-mediated apoptosis of oesophageal cancer cells (Sutter *et al*, 2002). In other cell types, a mitochondria-dependent mechanism has been suggested (Fischer *et al*, 2001; Maaser *et al*, 2001). Additionally, PBR-ligand-induced apoptosis of thymocytes was found to be sensitive to actinomycin D, cycloheximide, and the protein kinase inhibitor H7, suggesting a requirement for protein synthesis and phosphorylation (Tanimoto *et al*, 1999). Downregulation of Bcl-2 expression and dephosphorylation of protein kinase B and Bad were shown to be associated with PBR-ligand-induced apoptosis of hepatic stellate cells (Fischer *et al*, 2001). However, little is known about other transcriptional responses to treatment with PBR-specific ligands.

Cell cycle regulators are frequently disabled in human cancer. Hence, the modulation of cell cycle regulation is a feasible strategy for treating cancer (Shapiro and Harper, 1999). The induction of cell cycle arrest is a well-studied property of PBR-specific ligands. In oesophageal cancer cells, PBR-specific ligands induce a tumour-selective growth arrest at the G1/S checkpoint (Sutter *et al*, 2002). Likewise, a PBR-ligand-induced G1/S arrest was found in colorectal cancer cells (Maaser *et al*, 2001). Additionally, PBR ligands induce a cell cycle arrest at both major restriction points, the G1/S- and the G2/M junction (Carmel *et al*, 1999; Sa"nger *et al*, 2000) in breast cancer cells, whereas in lung and melanoma cells an accumulation is observed in the G2/M phase (Landau *et al*, 1998). However, the exact mechanism of PBR-ligand-mediated cell cycle arrest is not yet understood.

Employing cDNA arrays and RT–PCR, we assessed the PBR-ligand-induced downstream transcriptional profiles in oesophageal cancer cells. Here, we report on p38MAPK activation and *gadd* overexpression in response to PBR-specific ligands, leading to apoptosis and cell cycle arrest.

## MATERIAL AND METHODS

### Cell lines and drugs

The human oesophageal squamous carcinoma cell line KYSE-140 (Shimada *et al*, 1992) and the human colorectal adenocarcinoma cell line HT-29 were grown in RPMI 1640 medium supplemented with 10% FBS. The human oesophageal adenocarcinoma cell line OE-33 (Rockett *et al*, 1997) was grown in RPMI 1640 medium supplemented with 10% FBS and 2 mM L-glutamine. Cell lines were cultured in a humidified atmosphere (5% CO_2_) at 37°C. To evaluate the effects of PBR-specific ligands, cells were incubated with either control medium or medium containing FGIN-1-27, PK 11195 (Tocris, Bristol, UK), clonazepam (Sigma, Deisenhofen, Germany), or FGIN-1-52 (Kozikowski *et al*, 1993). SB202190 (2–20 *μ*M, Calbiochem-Novabiochem, Bad Soden, Germany) was used for p38MAPK inhibition. DEVD-CHO (10 *μ*g ml^−1^, Calbiochem-Novabiochem, Bad Soden, Germany) was used for caspase-3 inhibition.

### RNA extraction and polyA^+^ mRNA preparation

Total RNA was extracted from cultured cell lines with RNAClean following the recommendation of the manufacturer (Hybaid, London, UK). Polyadenylated (polyA^+^) mRNAs were enriched using magnetic Dynabeads according to the instructions of the supplier (Dynal, Oslo, Norway). The quality of polyA^+^ and total RNA was controlled by agarose gel electrophoresis.

### cDNA array

KYSE-140 and OE-33 cells were treated with FGIN-1-27 (50 *μ*M) for 24 h to determine PBR-ligand-induced differential gene expression. Untreated cells served as controls. We used an Atlas Human Apoptosis cDNA array with 205 human cDNAs spotted in duplicate on a nylon membrane (Clontech, Palo Alto, CA, USA). A complete list of the cDNAs and controls as well as their accession numbers is available on the web (http://atlasinfo.clontech.com/ genelists/huApop.xls). The membranes were hybridised with labelled cDNA probes prepared by reverse transcription from 1 *μ*g polyA^+^ mRNA using the respective protocol from Clontech. Probes used for hybridisation were equalised to a radioactivity of 2 × 10^6^ cpm ml^−1^ hybridisation solution. Overnight incubation was followed by stringent washing as recommended by the manufacturer. The membranes were then exposed to X-ray film for quantification. The hybridisation signals were photometrically evaluated using TINA software (raytest Isotopenmessgeräte, Straubenhardt, Germany).

### cDNA array data analysis

Alteration in the expression of a respective gene is given as fold increase/or decrease compared to the signal of the untreated control (Höpfner *et al*, 2002). For determining up- and downregulation, the mean optical density ×  mm^−2^ (OD  × mm^−2^) of each gene was identified and normalised to the expression of different housekeeping genes (ubiquitin, glyceraldehyde 3-phosphate dehydrogenase (GAPDH), tubulin *α* 1 subunit, HLA class I histocompatibility antigen C-4 *α* subunit (HLAC), cytoplasmic *β*-actin, 60S ribosomal protein L13A, 40S ribosomal protein S9). Then the ratio of gene expression in treated *vs* untreated cells was calculated. Data were used only when both signals were 50% or more above background, and if the deviations between duplicates did not exceed the difference between treated and untreated conditions. Each hybridisation experiment was repeated three times.

### Semiquantitative RT–PCR

Semiquantitative analysis of mRNA expression of the genes coding for *gadd45*, *gadd153*, and for the housekeeping gene *β*-actin was carried out by RT–PCR with the number of cycles at which the band intensity increased linearly with the amount of mRNA used. For RT–PCR, 2 *μ*g of total RNA was digested with 1 U DNAse I (Gibco, Karlsruhe, Germany) for 15 min at room temperature. Oligo-dT-primers and the SuperScript Preamplification-Kit (Gibco, Karlsruhe, Germany) were used for cDNA synthesis. PCR reactions were carried out in a total volume of 50 *μ*l containing 400 nM of each primer, 200 *μ*M of each dNTP (Pharmacia, Uppsala, Sweden), 50 mM KCl, 1.5 mM MgCl_2_, 10 mM Tris, and 1 U *Taq*-Polymerase (Pharmacia, Uppsala, Sweden). PCR was performed in a Peltier thermocycler (PTC-200, MJ-Research, USA) with the primers and at the conditions indicated in Table 1

**Table 1:** Primer sequences and PCR conditions used to evaluate the expression of the transcripts indicated

**Genes**	**Primers (5′–3′)**	**Position in the mRNA**	**Product size (bp)**	**Denaturing temperature and time (s)**	**Annealing temperature and time (s)**	**Extension temperature and time (s)**	**Number of cycles**
gadd45	F: AGAACGACATCAACATCCTGC	534–554	144	95°C (30)	60°C (30)	72°C (60)	35
	R: AATGTGGATTCGTCACCAGCA	657–677					
gadd153	F: AACCAGCAGAGGTCACAAGC	377–396	217	95°C (30)	60°C (30)	72°C (60)	33
	R: AGCCGTTCATTCTCTTCAGC	574–593					
β-actin	F: ATCATGTTTGAGACCTTCAACAC	437–459	822	94°C (40)	63°C (60)	72°C (60)	29
	R: TCTGCGCAAGTTAGGTTTTGTC	1237–1258					

All templates were initially denatured for 5 min at 95°C and the amplicon was extended at a final extension temperature of 72°C for 7 min.

(Oh-Hashi *et al*, 2001).

### Western blotting

Whole-cell extracts were prepared by harvesting and lysing the cells with lysis buffer (SDS 0.1%, sodium deoxycholic acid 0.5%, Nonidet P-40 1%, PMSF 0.1 mM, aprotinin 1 *μ*g ml^−1^, pepstatin A 1 *μ*g ml^−1^). The protein content of the lysate was determined using the BCA protein assay kit (Pierce, Rockford, IL, USA). The cell lysate was mixed with gel loading buffer (Tris-HCl 62.5 mM, glycerol 10%, SDS 1%, *β*-mercaptoethanol 2.5%). After boiling for 5 min, the lysates were subjected to SDS–polyacrylamide gel electrophoresis (20 *μ*g of protein per lane; gel: polyacrylamide 12%, SDS 0.1%, Tris-HCl 25 mM; running buffer: Tris 25 mM, glycine 50 mM, 0.1% SDS). After electrophoresis, gels were equilibrated with transfer buffer (Tris 25 mM, glycine 50 mM, 20% methanol). Proteins were transferred to PVDF membranes by electroblotting. Blots were blocked in 1.5% BSA, and then incubated at 4°C overnight with anti-human p38MAPK or phospho-p38MAPK (1 : 500, Santa Cruz Biotechnology, CA, USA). After washing with PBS containing 0.1% Tween and incubation with horseradish peroxidase-coupled anti-IgG antibody (1 : 10000, Amersham, Uppsala, Sweden) at room temperature for 1 h, the blot was washed extensively and developed using enhanced chemiluminescent detection (Amersham, Uppsala, Sweden). Blots were exposed to Hyperfilm ECL film (Amersham, Uppsala, Sweden) for 1–30 min and analysed densitometrically using TINA software (raytest Isotopenmessgeräte, Straubenhardt, Germany).

### Caspase-3 activity assay

To determine caspase-3 activity, cells were washed twice with PBS and stored at −80°C until use. Cells (10^6^) were lysed with lysis buffer (Tris-HCl 10 mM, NaH_2_PO_4_/Na_2_HPO_4_ 10 mM, NaCl 130 mM, Triton X-100 1%, NaPP_i_ 10 mM, pH 7.5), and the total protein content was quantified using the BCA protein assay kit (Pierce, Rockford, IL, USA). The activity of caspase-3 was calculated from the cleavage of the fluorogenic substrate DEVD-AMC (Calbiochem-Novabiochem, Bad Soden, Germany). In brief, cell lysates were incubated with substrate solution (caspase-3 substrate AC-DEVD-AMC 20 *μ*g ml^−1^, HEPES 20 mM, glycerol 10%, DTT 2 mM, pH 7.5) for 1 h at 37°C. The cleavage of DEVD-AMC was measured with a VersaFluor fluorometer (excitation: 360 nm emission: 460 nm) from Biorad, Munich, Germany (Maaser *et al*, 2002b).

### DNA fragmentation

DNA fragmentation was determined by Cell Death Detection ELISA (Roche Molecular Biochemicals) according to the manufacturer's instructions (Höpfner *et al*, 2003). Briefly, after incubation with the indicated compounds, cells were lysed in incubation buffer. The cytoplasmic fractions were diluted to contain 2.5 × 10^3^ cell equivalents per ml, and presence of mono- and oligonucleosomes was tested using antibodies directed against DNA and histones. DNA fragments were detected by a peroxidase system, with colour development analysed at 405 nm by an ELISA reader.

### Cell cycle analysis

Cell cycle analysis was performed by the method of Vindelov and Christensen (1990). Cells were trypsinised, washed, and the nuclei were isolated using CycleTest PLUS DNA Reagent Kit (Becton Dickinson, Heidelberg, Germany). DNA was stained with propidium iodide according to the manufacturer's instructions. The DNA content of the nuclei was detected by flow cytometry and analysed using CellFit software (Becton Dickinson, Heidelberg, Germany).

### Statistical analysis

Individual drug therapy was compared by the unpaired, two-tailed Mann–Whitney *U*-test. The unpaired Student's *t*-test was used for cell cycle analysis. *P*-values were considered to be significant at <0.05. If not stated otherwise, all experiments were performed in quadruplicate.

## RESULTS

### Peripheral benzodiazepine receptor-ligand-induced differential gene expression

Peripheral benzodiazepine receptor-specific ligands have been shown to induce apoptosis and cell cycle arrest potently in human oesophageal cancer cells (Sutter *et al*, 2002). We used cDNA arrays to analyse changes in the expression of apoptosis- and cell cycle-regulating genes elicited by PBR-specific ligands (Höpfner *et al*, 2002). For cDNA array experiments, we chose a concentration of 50 *μ*M FGIN-1-27 and a 24-h incubation time, which is sufficient to induce apoptosis and cell cycle arrest (Maaser *et al*, 2001; Sutter *et al*, 2002).

We found over 45 genes that were differentially expressed in both KYSE-140 and OE-33 cells. FGIN-1-27 treatment resulted in an asymmetric distribution of overexpressed *vs* suppressed genes. Moreover, a comparison of the up- and downregulated genes revealed discrepancies in the FGIN-1-27-induced regulation between the two cell lines: in KYSE-140 (12 overexpressed, 35 suppressed), 25.5% of the regulated genes were overexpressed and 74.5% were suppressed. In contrast, 65.2% of the genes were overexpressed and 34.8% were suppressed in OE-33 cells (30 overexpressed, 16 suppressed). Tables 2

**Table 2:** Transcripts differentially regulated in KYSE-140 in response to FGIN-1-27

**GenBank ID**	**Gene name**	**Mean**^a^	**s.d.**
S40706	gadd153	7.24	2.73
X96586	FAN protein	3.70	0.07
X07282	Retinoic acid receptor, *β*	2.63	0.82
X08020	Glutathione *S*-transferase M4	2.49	0.98
M60974	gadd45	2.27	0.88
U34051	Cyclin-dependent kinase 5	1.88	0.30
L26318	Mitogen-activated protein kinase 8, JNK1	1.63	0.04
U76376	Harakiri. BCL2-interacting protein	1.59	0.13
L29220	CDC-like kinase 3	1.57	0.42
X15480	Glutathione *S*-transferase pi	1.46	0.07
U11791	Cyclin H	1.45	0.21
M84820	Retinoid X receptor, beta	1.23	0.17
M25627	Glutathione *S*-transferase A2	0.82	0.15
X03484	v-raf-1 murine leukaemia viral oncogene homolog 1	0.78	0.11
Y00285	Insulin-like growth factor 2 receptor	0.77	0.08
M62402	Insulin-like growth factor binding protein 6	0.77	0.13
L35253	Mitogen-activated protein kinase 14, p38MAPK	0.76	0.13
U37448	Caspase 7	0.75	0.01
S90469	*P*450 (cytochrome) oxidoreductase	0.75	0.03
D13639	Cyclin D2	0.74	0.10
AF016268	Tumour necrosis factor receptor superfamily, member 10b	0.74	0.10
M34065	Cell division cycle 25C	0.73	0.08
L16785	NM23B	0.73	0.17
X89986	BCL2-interacting killer (apoptosis-inducing)	0.71	0.12
U66879	BCL2-antagonist of cell death	0.71	0.06
U21092	TNF receptor-associated factor 3	0.66	0.11
U01038	Polo (Drosophia)-like kinase	0.65	0.16
X60188	Mitogen-activated protein kinase 3, ERK1	0.65	0.18
Y11416	Tumour protein p73	0.65	0.25
U34819	Mitogen-activated protein kinase 10, JNK3	0.64	0.03
L41690	TNFRSF1A-associated via death domain	0.64	0.28
AF015956	Death-associated protein 6	0.63	0.31
AF022385	Programmed cell death 10	0.62	0.15
U49070	Protein (peptidyl-proly∣*cis*/*trans* isomerase) NIMA-interacting 1	0.61	0.07
M84489	Mitogen-activated protein kinase 1, ERK2	0.58	0.06
X86779	Fas-activated serine/threonine kinase	0.58	0.17
U60520	Caspase 8	0.57	0.23
U90313	Glutathione transferase omega	0.56	0.16
M35410	Insulin-like growth factor binding protein 2	0.56	0.06
L22474	BCL2-associated X protein	0.55	0.15
U38545	Phospholipase D1	0.54	0.05
U10564	Wee1+ (*S*. *pombe*) homologue	0.49	0.13
D89667	Prefoldin 5	0.49	0.22
U82938	CD27-binding (Siva) protein	0.48	0.03
M74091	G1/S-specific cyclin C	0.44	0.22
M15796	Proliferating cell nuclear antigen	0.44	0.33
D38122	Tumour necrosis factor (ligand) superfamily, member 6	0.18	0.01

a Arithmetic means of ratios (treated: untreated) from three separate array measurements

and 3

**Table 3:** Transcripts differentially regulated in OE-33 in response to FGIN-1-27

**GenBank ID**	**Gene name**	**Mean**^a^	**s.d.**
S40706	gadd153	2.76	0.82
X60188	Mitogen-activated protein kinase 3, ERK1	2.02	0.45
U90313	Glutathione transferase omega	1.94	0.86
L27211	Cyclin-dependent kinase inhibitor 2A	1.92	0.68
X59798	Cyclin D1	1.91	0.77
M81934	Cell division cycle 25B	1.88	0.39
M31159	Insulin-like growth factor binding protein 3	1.87	0.62
U34051	Cyclin-dependent kinase 5	1.83	0.37
M63167	v-akt murine thymoma viral oncogene homolog 1	1.83	0.36
U28014	Caspase 4	1.82	0.31
U63131	Cell division cycle 37	1.74	0.46
Y00285	Insulin-like growth factor 2 receptor	1.69	0.05
S78085	Programmed cell death 2	1.68	0.04
M34065	Cell division cycle 25C	1.67	0.63
AF010312	LPS-induced TNF-alpha factor	1.67	0.21
L05624	Mitogen-activated protein kinase kinase 1, MEK1	1.62	0.45
L41690	TNFRSF1A-associated via death domain	1.59	0.37
M1*45*05	Cyclin-dependent kinase 4	1.57	0.35
L29511	Growth factor receptor-bound protein 2	1.56	0.38
D89667	Prefoldin 5	1.54	0.25
U82938	CD27-binding (Siva) protein	1.54	0.50
L29216	CDC-like kinase 2	1.53	0.43
M15796	Proliferating cell nuclear antigen	1.48	0.32
M84820	Retinoid X receptor, beta	1.48	0.31
X96586	FAN protein	1.44	0.23
L22005	Cell division cycle 34	1.40	0.18
U13737	Caspase 3	1.38	0.26
M60974	gadd45	1.37	0.00
X92669	Menage a trois 1 (CAK assembly factor)	1.33	0.05
M81933	Cell division cycle 25A	1.23	0.11
M29645	Insulin-like growth factor 2 (somatomedin A)	0.89	0.05
X01394	Tumour necrosis factor (TNF superfamily, member 2)	0.83	0.03
X15480	Glutathione *S*-transferase pi	0.77	0.05
L07414	Tumour necrosis factor (ligand) superfamily, member 5	0.75	0.19
U69108	TNF receptor-associated factor 5	0.72	0.05
D12614	Lymphotoxin alpha (TNF superfamily, member 1)	0.72	0.12
U33286	Chromosome segregation 1 (yeast homologue)-like	0.70	0.04
U78798	TNF receptor-associated factor 6	0.67	0.04
L08246	Myeloid cell leukaemia sequence 1 (BCL2-related)	0.62	0.10
M73812	Cyclin E1	0.61	0.16
U56390	Caspase 9	0.59	0.19
U60520	caspase 8	0.55	0.34
L31951	Mitogen-activated protein kinase 9, JNK2	0.48	0.50
X05360	Cell division cycle 2	0.44	0.07
U75285	Survivin	0.43	0.25
M32315	Tumour necrosis factor receptor superfamily, member 1B	0.41	0.31

a Arithmetic means of ratios (treated : untreated) from three separate array measurements

show the genes regulated by FGIN-1-27 in KYSE-140 and OE-33 cells, respectively.

Tanimoto *et al* (1999) have demonstrated that PBR-ligand-induced apoptosis required protein *de novo* synthesis. Therefore, we focused on the genes being overexpressed in both the cancer cell lines. In KYSE-140, five genes were induced by FGIN-1-27 to a level exceeding the expression ratio of 2.0. Three of these genes (*gadd153*, *gadd45*, and factor associated with neutral sphingomyelinase activation) were also induced in OE-33 cells, suggesting that these genes are involved in a common signalling pathway. *gadd45* and *gadd153*, both of which are activated by p38MAPK (Kultz *et al*, 1998), have been associated with apoptosis and growth arrest induced by various extracellular stimuli (Kultz *et al*, 1998; Maytin *et al*, 2001; Oh-Hashi *et al*, 2001). These two genes were selected for further analysis.

### Peripheral benzodiazepine receptor-specific ligands induce *gadd45* and *gadd153* mRNA expression

**Figure 1:**
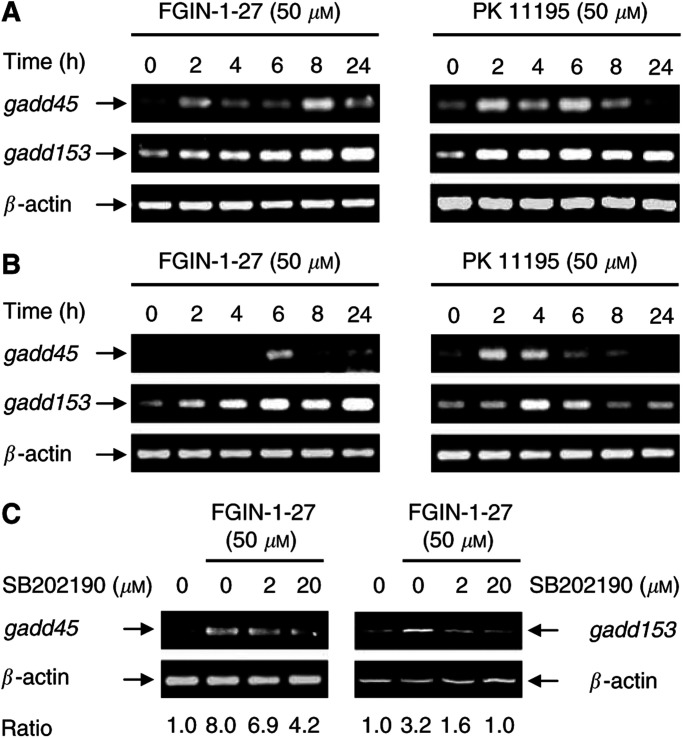
mRNA expression of *gadd45* and *gadd153* in response to PBR-ligands: involvement of the p38MAPK signalling pathway. mRNA expression of *gadd45* and *gadd153* in KYSE-140 cells (**A**) or OE-33 cells (**B**) was detected after incubation with FGIN-1-27 or PK 11195. (**C**) mRNA expression of *gadd45* and *gadd153* in KYSE-140 cells treated with FGIN-1-27 for 8 h in the presence or absence of SB202190. Pretreatment with SB202190 markedly reduced *gadd* induction elicited by FGIN-1-27.

Semiquantitative RT–PCR analysis was performed to confirm the overexpression of *gadd45* and *gadd153* observed by cDNA array analysis and to monitor their temporal induction. FGIN-1-27 and PK 11195 induced *gadd45* and *gadd153* rapidly after 2–4 h of treatment, with maximal expression of both transcripts occurring after 8–24 h for FGIN-1-27 and after 4–6 h for PK 11195. The kinetics of induction, however, differed for the two ligands: during treatment with PK 11195, both transcripts returned to basal level after 8 h, but remained elevated up to 24 h after treatment with FGIN-1-27 (Figure 1A, B

). To study if *gadd* overexpression commonly occurred in response to PBR activation, we also analysed *gadd* expression in FGIN-1-27- or PK 11195-treated HT-29 colorectal cancer cells. HT-29 cells have previously been characterised regarding PBR expression and PBR-ligand-induced apoptosis (Maaser *et al*, 2001). Both FGIN-1-27 and PK 11195 induced a transient overexpression of *gadd153*, reaching a maximal induction after 24 h (50 *μ*M FGIN-1-27, ratio=7.0±3.2) or 6 h (50 *μ*M PK 11195, ratio=3.3±0.4). Similar to the findings in oesophageal cancer cells, *gadd45* was also overexpressed in HT-29 cells after a 24-h incubation with PBR ligands (data not shown).

### p38MAPK activation contributes to *gadd45* and *gadd153* induction

Mitogen-activated protein (MAP) kinases represent one of the most important signalling cascades in response to extracellular stimuli (Chan-Hui and Weaver, 1998). To gain an insight into the PBR-ligand-mediated signal transduction pathways responsible for *gadd45* and *gadd153* induction, we determined the influence of the p38MAPK (stress-activated protein kinase 2) cascade. We used the potent p38MAPK inhibitor SB202190 (Herlaar and Brown, 1999; Lee *et al*, 2000; Mayr *et al*, 2002) to determine whether p38MAPK activation is directly associated with the induction of *gadd* messages in oesophageal cancer cells. SB202190 belongs to a family of pyridinyl imidazole compounds that have been shown to inhibit specifically p38MAPkinase activity at the concentrations used, but do not exhibit any significant effect upon a variety of other kinases such as JNK, ERK-1, and MAPKAP kinase 2 (Lee *et al*, 1994; Cuenda *et al*, 1995). The FGIN-1-27-mediated induction of *gadd45* and *gadd153* transcripts was markedly decreased after preincubating the cells with SB202190 for 1 h (Figure 1C). SB202190 alone had no effect on *gadd* expression (data not shown). These data suggest that p38MAPK activation contributes to the induction of *gadd45* and *gadd153* by the PBR-specific ligand FGIN-1-27.

### p38MAPK activation by PBR-specific ligands

**Figure 2:**
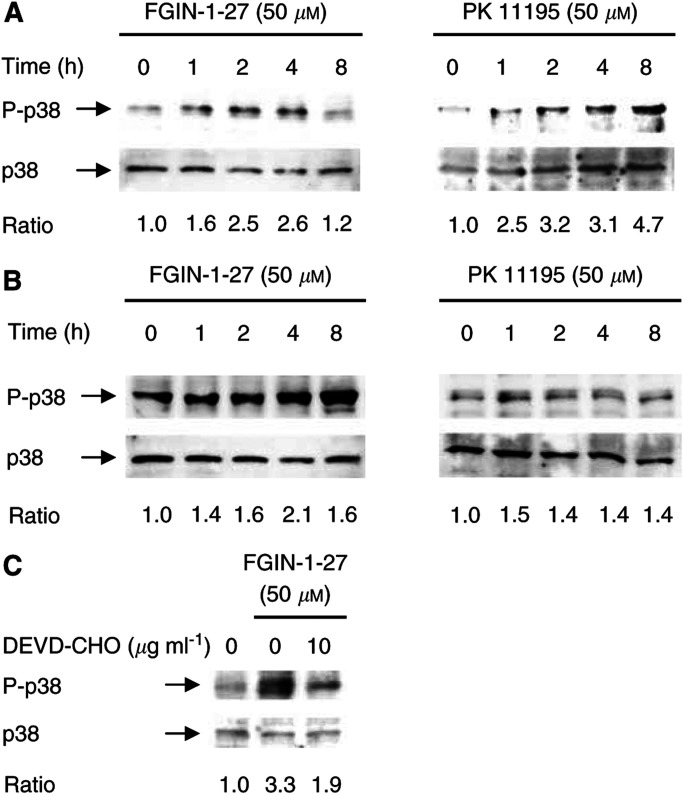
Activation of p38MAPK by PBR-specific ligands: involvement of caspase-3. Posphorylation of p38MAPK was analysed by Western blotting using antibodies against the active form (P-p38MAPK) and nonphosphorylated p38MAPK. p38MAPK was transiently phosphorylated in KYSE-140 (**A**) and OE-33 cells (**B**) by FGIN-1-27 or PK 11195. (**C**) p38MAPK phosphorylation in KYSE-140 cells treated with either vehicle, FGIN-1-27 (4 h), or DEVD-CHO for 1 h followed by FGIN-1-27 for 4 h. Pretreatment with DEVD-CHO attenuated FGIN-1-27-induced p38MAPK phosphorylation.

Phosphorylation-mediated activation of the p38MAPK by PBR-specific ligands was determined by Western blotting. Both PBR-specific ligands, FGIN-1-27 and PK 11195, induced a time- and dose-dependent phosphorylation of p38MAPK, thereby showing high correlation with the induction of *gadd* transcripts (Figure 2A, B

). The maximum of p38MAPK activation was observed after 4 h (FGIN-1-27) or 1–8 h (PK 11195) of treatment. After 4 h, we observed an about 1.7-fold activation of p38MAPK in response to 10 *μ*M of the respective PBR ligand and an about 3.1-fold activation in response to 50 *μ*M of either ligand. At 100 *μ*M, we detected an up to 4.1-fold increase of activated p38MAPK. As activation was pronounced at 50 *μ*M of either ligand already, we chose this concentration for further experiments. PBR-specific ligands did not affect the expression of either p38MAPK protein (Figure 2) or mRNA (data not shown) analysed within a period of 24 h.

### FGIN-1-27-induced caspase-3 activation contributes to activation of p38MAPK

**Figure 3:**
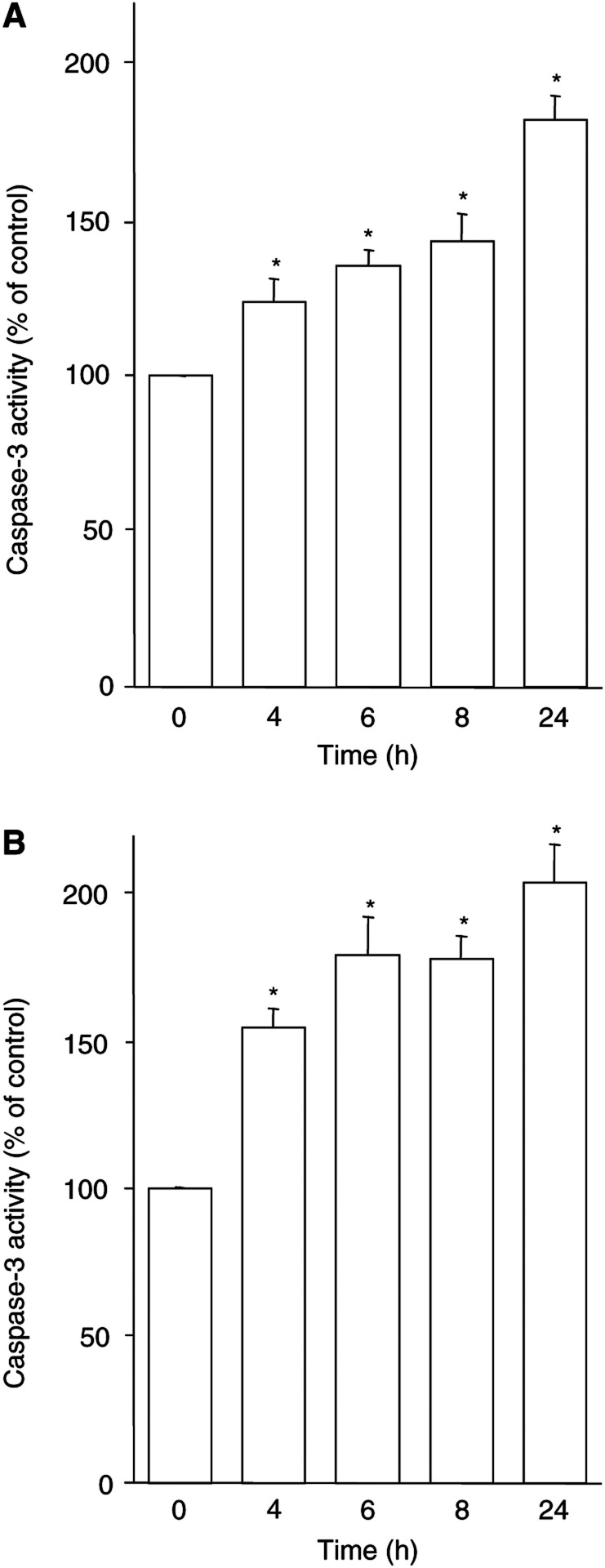
PBR-ligand-induced caspase-3 activation. KYSE-140 (**A**) or OE-33 cells (**B**) were treated with FGIN-1-27 (50 *μ*M). Caspase-3 activity was measured fluorometrically by the cleavage of DEVD-AMC. Data are given as percentage of untreated control (means±s.e.m. of four independent experiments). ^*^ Statistical significance (*P*<0.05) compared to untreated control.

Peripheral benzodiazepine receptor-ligand-mediated apoptosis involves caspase-3 activation, leading to DNA fragmentation and cell death (Sutter *et al*, 2002). The PBR-specific ligand FGIN-1-27 induced a dose- (Sutter *et al*, 2002) and time-dependent increase in caspase-3 activity in both KYSE-140 (Figure 3A

) and OE-33 (Figure 3B) cells. The time course of PBR-ligand-induced caspase-3 activation correlates with p38MAPK phosphorylation, suggesting a link between caspase-3 and p38MAPK activation. Thus, a possible regulatory relationship between caspase-3 and p38MAPK during PBR-ligand-mediated apoptosis was investigated. KYSE-140 cells were treated with FGIN-1-27 (50 *μ*M) for 4 h in the presence or absence of the caspase-3 inhibitor DEVD-CHO (10 *μ*g ml^−1^). This concentration was previously shown to be sufficient to inhibit caspase-3 activation and DNA fragmentation (Sutter *et al*, 2002). Cells pretreated with DEVD-CHO displayed a markedly (−42%) reduced activation of p38MAPK (Figure 2C), indicating that in FGIN-1-27-induced apoptosis of KYSE-140 cells, caspase-3 activation contributes to p38MAPK activation. On the other hand, pretreatment of KYSE-140 cells with SB202190 did not prevent caspase-3 activation by FGIN-1-27, even at the highest concentration of the p38MAPK inhibitor, demonstrating that p38MAPK activation is not required for caspase-3 activation (data not shown).

### FGIN-1-27-induced p38MAPK activation contributes to DNA fragmentation

**Figure 4:**
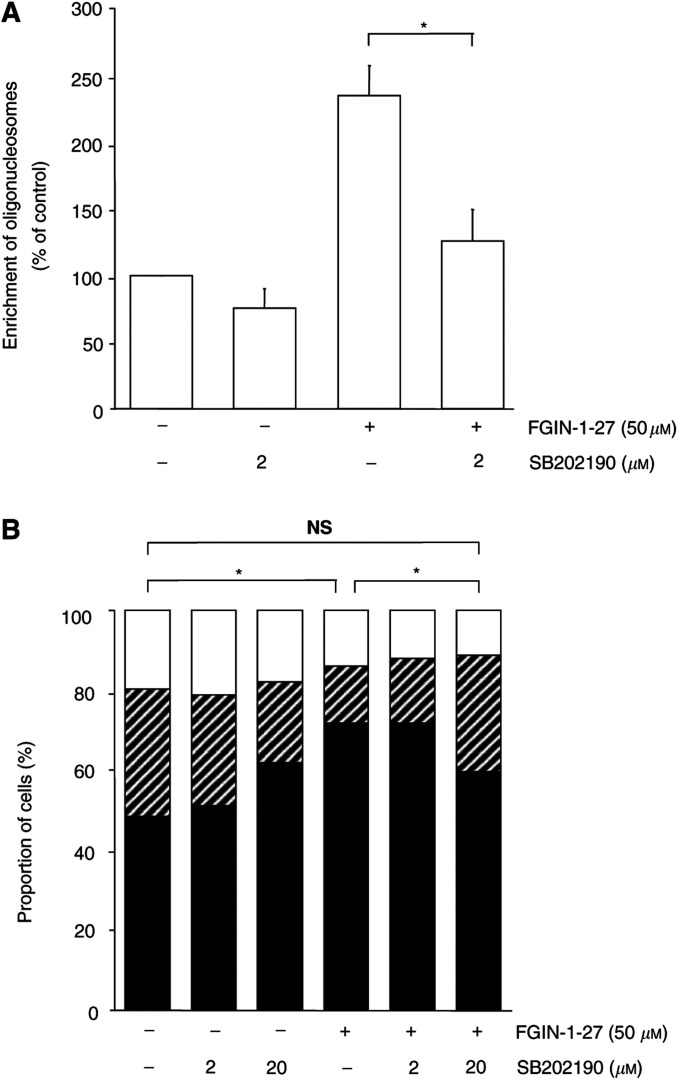
p38MAPK activation is required for FGIN-1-27-mediated DNA fragmentation and G1/S arrest. (**A**) KYSE-140 cells, incubated with 50 *μ*M FGIN-1-27 alone or in combination with SB202190, were analysed for DNA fragmentation. Cells were preincubated for 1 h with SB202190 prior to FGIN-1-27 addition and subsequent incubation for 14 h. Untreated cells (ctrl), and cells treated only with SB202190, were also run as controls. SB202190 strongly diminished FGIN-1-27-induced DNA fragmentation. (**B**) KYSE-140 cells were treated with either vehicle, FGIN-1-27 for 24 h, SB202190 for 25 h, or SB202190 for 1 h followed by FGIN-1-27 plus SB202190 for 24 h. Treatment with FGIN-1-27 increased the proportion of cells in the G0/G1 phase (black columns), whereas the proportion of cells in the S (hatched columns) and G2/M phases (white columns) decreased. Pretreatment with SB202190 abolished FGIN-1-27-mediated G1/S arrest. Means of three independent experiments±s.e.m are shown. ^*^P<0.05, NS=not significant.

Finally, we investigated if p38MAPK activation contributes to FGIN-1-27-induced DNA fragmentation. Thus, KYSE-140 cells were treated with FGIN-1-27 (50 *μ*M) either in the presence or in the absence of SB202190. Cells pretreated with SB202190 for 1 h displayed a reduced DNA fragmentation, providing evidence that p38MAPK activation is involved in DNA fragmentation (Figure 4A

).

### Activation of the p38MAPK pathway is responsible for FGIN-1-27-mediated cell cycle arrest

Peripheral benzodiazepine receptor ligands have been shown to arrest the cell cycle of human oesophageal cancer cells in the G0/G1 phase, thereby causing a G1/S arrest (Sutter *et al*, 2002). Thus, we analysed whether p38MAPK activation was involved in PBR-ligand-mediated cell cycle arrest. Preincubating KYSE-140 cells with SB202190 completely abolished FGIN-1-27-induced G1/S arrest in oesophageal cancer cells (Figure 4B), suggesting that p38MAPK activation is required for the cell cycle arrest observed. SB202190 alone did not have any significant impact on the cell cycle.

## DISCUSSION

In this study we provide an insight into the signal transduction pathway by which PBR-specific ligands induce cell cycle arrest and apoptosis in oesophageal cancer cells. Peripheral benzodiazepine receptor-specific ligands activate the p38MAPK signalling pathway, leading to overexpression of *gadd45* and *gadd153* and cell cycle arrest. Furthermore, we show that PBR-ligand-induced caspase-3 activation contributes to p38MAPK activation, resulting in DNA fragmentation. This suggests an involvement of p38MAPK in PBR-mediated apoptosis.

The p38MAPK pathway is known to be activated by a variety of stimuli including UV irradiation, hydrogen peroxide, DNA damage, heat, and hyperosmotic shock. Activation of the p38MAPK pathway results in growth arrest and apoptosis (Kultz *et al*, 1998; Ichijo, 1999). We used the pyridinyl imidazole inhibitor SB202190 to demonstrate the involvement of p38MAPK in PBR-ligand-mediated apoptosis and cell cycle arrest. SB202190 has been shown to inhibit p38MAPK *α* and *β* at the concentrations applied, whereas it shows no effect against a large panel of other related protein kinases tested (Davies *et al*, 2000). SB202190 has widely been used to study the involvement of p38MAPK in proliferation, apoptosis, and differentiation (Kultz *et al*, 1998; Oh-Hashi *et al*, 2001). In this study, SB202190 prevented PBR-ligand-induced apoptosis and G1/S arrest, suggesting an involvement of the p38MAPK pathway.

Mitogen-activated protein kinase (MAPK) signal transduction pathways are known to regulate the expression of the *gadd* genes (Kultz *et al*, 1998; Oh-Hashi *et al*, 2001). Thus, our next goal was to elucidate if p38MAPK is involved in PBR-ligand-mediated *gadd* overexpression. The expression of the *gadd45* gene has been correlated with the presence of strong growth arrest (Zhan *et al*, 1994a), and it has been shown to associate with proliferating cell nuclear antigen (PCNA), where it may play a role in DNA repair (Smith *et al*, 1994). Overexpression of each *gadd* gene causes growth inhibition and/or apoptosis, and combined overexpression of the *gadd* genes leads to a synergistic suppression of cell growth (Zhan *et al*, 1994b). In this study, inhibition of p38MAPK activity by SB202190 suppressed the expression of *gadd* genes induced by PBR-specific ligands. These results confirm earlier findings that *gadd* induction occurs as a direct consequence of p38MAPK activation (Oh-Hashi *et al*, 2001). The potency of SB202190 to inhibit *gadd45* and *gadd153* overexpression correlated well with its ability to decrease apoptosis and cell cycle arrest, suggesting an involvement of *gadd* genes in apoptosis and G1/S arrest. In accordance with our findings, it has been reported that G1/S arrest is a result of *gadd* induction by p38MAPK (Smith *et al*, 1994). However, as even the highest concentration of SB202190 only partially prevented the increase in *gadd45* and *gadd153* expression, other still unidentified, p38MAPK-independent pathways (Maytin *et al*, 2001) may contribute to PBR-ligand-mediated *gadd* induction: the generation of reactive oxygen species, the activation of the p53 pathway or the JNK pathway are well known to induce *gadd* genes, too (Guyton *et al*, 1996; Sheikh *et al*, 2000). In our previous report on PBR-ligand-mediated apoptosis of oesophageal cancer cells, we showed that disruption of the mitochondrial membrane potential was required for caspase-3 activation (Sutter *et al*, 2002). Using the caspase-3-specific inhibitor DEVD-CHO, we now demonstrate that caspase activation is upstream of p38MAPK activation, supporting previous findings in Jurkat T lymphocytes (Juo *et al*, 1997, 1998; Matsuda *et al*, 1999). Interestingly, caspase-3 activation occurs independently of p38MAPK, as the p38MAPK inhibitor SB202190 failed to prevent the activation of caspase-3. Furthermore, FGIN-1-27-induced DNA fragmentation is decreased by pretreatment with SB202190, suggesting an involvement of p38MAPK in PBR-ligand-mediated formation of oligonucleosomes.

As p38MAPK activation also proved to be a prerequisite for PBR-ligand-mediated cell cycle arrest, we provide a link between PBR-ligand-mediated induction of apoptosis and G1/S arrest. Furthermore, we extend our current model on PBR-ligand-mediated apoptosis. The signalling cascade comprises mitochondrial membrane permeabilisation leading to caspase-3 activation, followed by p38MAPK activation and finally DNA fragmentation.

To clarify the PBR specificity of the effects observed, we applied FGIN-1-52, a structural analogue of FGIN-1-27, and the benzodiazepine clonazepam; neither substance binding to PBR (Wang *et al*, 1984b; Kozikowski *et al*, 1993). Clonazepam or FGIN-1-52 did not affect p38MAPK phosphorylation, *gadd* expression (data not shown), apoptosis, or the cell cycle (Maaser *et al*, 2001; Sutter *et al*, 2002). This indicates that the dose- and time-dependent induction of the p38MAPK signalling pathway by FGIN-1-27 and PK 11195 is PBR specific.

Protein *de novo* synthesis was shown to be required for PBR-ligand-mediated induction of apoptosis (Tanimoto *et al*, 1999). Therefore, we analysed the transcriptional changes in response to treatment with FGIN-1-27. Using an apoptosis- and cell-cycle-specific cDNA array spotted with 205 genes related to proliferation, apoptosis and cell cycle, we have identified 78 genes, about 45 genes in either cell line, responsive to FGIN-1-27 treatment. The fact that PBR-specific ligands exert antiproliferative effects in different tumours (Wang *et al*, 1984a; Maaser *et al*, 2001) suggests that PBR ligands interfere with a common signalling pathway. The expression patterns elicited by FGIN-1-27 partially overlapped between KYSE-140 and OE-33 cells, suggesting that those genes commonly regulated in both cell lines are of general importance for apoptosis and cell cycle arrest. Out of the 205 genes, only three of those exceeding an expression ratio of 2.0 in KYSE-140 cells were also overexpressed in OE-33 cells. Two of them were *gadd45* and *gadd153*, both of which are known to be transcriptionally regulated (Kultz *et al*, 1998). Our functional data indicate that *gadd45* and *gadd153* overexpression plays an important role in PBR-ligand-mediated apoptosis and cell cycle arrest. Many genes were regulated by FGIN-1-27 treatment in only one of the two cell lines. Apparently, cell-type-specific differences occur in the signalling pathways involved in the effects of FGIN-1-27. Moreover, differences between the two cell lines may also reflect differences in the cellular stress response to the initial stimulus. For example, in both the cell lines we found an overexpression of glutathione transferases in response to treatment with FGIN-1-27. However, different isoforms of the antioxidant enzyme were induced. In spite of the differences, the expression patterns of both cell lines after treatment with FGIN-1-27 reflect the apoptotic and growth-arrested phenotype of oesophageal cancer cells. In OE-33 cells, FGIN-1-27 treatment strongly decreased the expression of survivin, which is an antiapoptotic protein with prognostic relevance in oesophageal cancer (Grabowski *et al*, 2003). Furthermore, programmed cell death 2 protein (PDCD2), a gene associated with apoptosis of thymocytes (Kawakami *et al*, 1995), is induced by FGIN-1-27. The growth arrest is reflected in the overexpression of cdki2A and PCNA, the interaction partner of *gadd45*, and the downregulation of cdc2. In KYSE-140 cells, we observed a downregulation of cyclin C, cyclin D1 and cdc25A, all of which are associated with G1/S transition. However, the functional involvement of each protein has to be evaluated.

In summary, p38MAPK is dose- and time-dependently activated by PBR-ligands. Furthermore, *gadd* genes are overexpressed and apoptosis and cell cycle arrest are induced, all of which are known consequences of p38MAPK activation. Intriguingly, all effects can be antagonised by SB202190, which is described as a potent p38MAPK inhibitor. Thus, our data suggest that activating the p38MAPK pathway is a necessary step for inducing apoptosis and cell cycle arrest by PBR-specific ligands. Understanding the mechanisms of action will facilitate the design of combination chemotherapies that act additively or synergistically. Furthermore, some of the molecular targets like *gadd153* and *gadd45* might be used as surrogate biomarkers for future PBR-ligand intervention trials. Interestingly, using *gadd153* induction as a predictor of clinical response has already been evaluated for paclitaxel treatment of cancer patients (Las Alas *et al*, 2000). Hence, our data on the pathways responding to PBR-specific ligands, in combination with the knowledge that signalling pathways may be defective in tumours, will be helpful in predicting the responsiveness of tumours to PBR ligands in the future.
